# Chitosan Oligosaccharide Production Potential of *Mitsuaria* sp. C4 and Its Whole-Genome Sequencing

**DOI:** 10.3389/fmicb.2021.695571

**Published:** 2021-08-05

**Authors:** Duo Chen, Congcong Chen, Xuehai Zheng, Jiannan Chen, Wenjin He, Chentao Lin, Huibin Chen, Youqiang Chen, Ting Xue

**Affiliations:** The Public Service Platform for Industrialization Development Technology of Marine Biological Medicine and Products of the State Oceanic Administration, Fujian Key Laboratory of Special Marine Bioresource Sustainable Utilization, Key Laboratory of Developmental and Neural Biology, College of Life Sciences, Southern Institute of Oceanography, Fujian Normal University, Fuzhou, China

**Keywords:** *Mitsuaria* sp., chitosanase, purification, optimization, whole-genome sequencing

## Abstract

Chitooligosaccharide is a kind of functional food, which is the degradation product of chitosan (COS) catalyzed by the endo-chitosanase (COSE) enzyme. A COSE with a molecular weight of 34 kDa was purified and characterized from a newly isolated *Mitsuaria* sp. C4 (C4), and a 38.46% recovery rate and 4.79-fold purification were achieved. The purified C4 COSE exhibited optimum activity at 40°C and pH 7.2 and was significantly inhibited in the presence of Cu^2+^ and Fe^3+^. The *K*_*m*_ and *V*_*min*_ of the COSE toward COS were 2.449 g/L and 0.042 g/min/L, respectively. The highest COSE activity reached 8.344 U/ml after optimizing, which represented a 1.34-fold of increase. Additionally, chitooligosaccharide obtained by COSE hydrolysis of COS was verified by using thin-layer chromatography and high-performance liquid chromatography analysis. Whole-genome sequencing demonstrated that the C4 strain contains 211 carbohydrate enzymes, our purified COSE belonging to GHs-46 involved in carbohydrate degradation. Phylogenetic analysis showed that the novel COSE obtained from the C4 strain was clustered into the degree of polymerization = two to three groups, which can perform catalysis in a similar manner to produce (GlcN)_2_ and (GlcN)_3_. This work indicates that the C4 strain could be a good resource for enhancing carbohydrate degradation and might represent a useful tool for chitooligosaccharide production in the functional food industry.

## Introduction

Chitosan (COS) is a polysaccharide obtained by the deacetylation of chitin, and it is widely used in the food, material, medicine, agriculture, and chemical industries and environmental protection due to its special physiological and biochemical characteristics (Morin-Crini et al., 2019; Pandit et al., 2021; Yang et al., 2021). However, COS can only be dissolved in dilute hydrochloric acid, dilute nitric acid, and most organic acids, which significantly limits its application (Wu and Zhang, 2019). Chitooligosaccharide (COSS) is a basic amino oligosaccharide after partial hydrolysis of COS with a degree of polymerization (DP) of 2–20, and they are easily absorbed and utilized due to their low molecular weight, high water solubility, and biological activity (Chen et al., 2019). COSS production has attracted increasing attention for use in food and medicine because it can be used as functional food widely for improving immunity, inhibiting tumor cancer cell growth, and lowering blood pressure and blood sugar (Zhang et al., 2020). However, the COSS industry in China still faces a series of challenges, such as how to obtain high-purity COSS, how to control the molecular weight of COSS, and how to increase the actual yield. These problems have become bottlenecks that scientific researchers urgently need to solve.

Enzyme hydrolysis is the most popular method for producing COSS in the functional food industry, which occurs by the digestion of COS, and it has various advantages, such as high oligosaccharide yield and good reproducibility (Zhu et al., 2010; Jung and Park, 2014; Benchamas et al., 2021). Chitosanase (COSE, EC 3.2.1.132) is a specific glycosidase for the degradation of COS, and it can catalyze the hydrolysis of glycosidic bonds containing a glucosamine group (GlcN) and partially acetylated COS, which results in low molecular weight glycosidic COSS (Jiang et al., 2012; Li et al., 2021). Therefore, COSE can be effectively used to prepare COSS from COS substrates. Differences in COSE from different sources depend on the acetylation degree of the substrate and the enzymatic properties: (1) cleaving both GlcN-GlcN and GlcNAc-GlcN bonds (*Streptomyces* sp. N174) (Lacombe-Harvey et al., 2013); (2) cleaving only the GlcN-GlcN bond (*Bacillus* sp. MD-5) (Yang et al., 2020); and (3) cleaving both GlcN-GlcN and GlcN-GlcNAc bonds (Liu and Xia, 2006; Yabuki et al., 2006). COSE derived from mold, bacteria, and actinomycetes may be involved in isolation, purification, enzymatic mechanisms, catalytic properties, protein structure, and optimization of fermentation conditions (Lestari et al., 2020). COSE obtained from bacteria is widely used to maintain the balance of ecology and ecosystems, especially soil. Additionally, a number of studies are attempting to clone and express COSE genes. For example, COSE (SsCsn46) from *Streptomyces* sp. N174 was expressed in *Pichia pastoris* GS115 by deleting 198A, 199A, 200H, and 201D, thereby producing a COSS with DP > 4 and reducing enzyme activity (Ding et al., 2019). GHs-46 COSE (GsCsn46A) from *Gynuella sunshinyii* was cloned and expressed in *Escherichia coli*, resulting in three types of COSS products (DP 2–7, 2–5, and 2–3). Extracellular COSE from *Streptomyces* sp. N174 was expressed in *E. coli* and *Streptomyces lividans*. The results indicated that COSE is well expressed in *S. lividans* but poorly expressed in *E. coli* (Qin et al., 2018a). Although a large number of COSE genes have been extensively cloned and expressed, COS degradation products are usually characterized by diversity and instability. Hence, screening of high COSE-producing microorganisms represents an important scientific issue. Some COSE-producing *Mitsuaria* species have been reported to degrade COS, including *Mitsuaria* sp. 141-2 and *Mitsuaria chitosanitabida* 3001 (Yun et al., 2006). However, genes involved in COS degradation by *Mitsuaria* sp. still have not been widely cloned and expressed. Also, the characterization of COSE from *Mitsuaria* sp. at the genome-wide level is not clear yet.

In this study, the purification and properties of a newly isolated *Mitsuaria* sp. C4 (C4) COSE are reported. The properties of COSE, including the pH, temperature, metal ions, substrate specificity, kinetic constants, and hydrolyzate profiles, revealed that it is a novel and potential endo-type COSE for industrial applications. Statistical optimization of COSE production has been performed using single-factor experiments and the response surface methodology (RSM). To better understand the genomic basis of COSE activities and degradation function, whole-genome sequencing of C4 was performed. These results will provide a new useful tool for oligosaccharide production in the functional food industry.

## Materials and Methods

### Materials

Chitosan (degree of deacetylation >90%) was purchased from Henan Xingyuan Chemical Products Co., Ltd. (Henan, China). D-glucosamine was purchased from Sisco Research Laboratory (Mumbai, India). All other chemicals and reagents were of analytical grade and are available commercially. COS liquid medium (%, w/v) contained 1% COS, 0.2% KH_2_PO_4_, 0.1% NaCl, 0.1% KCl, 0.14% MgSO_4_.7H_2_O, 0.02% CaCl_2_, 0.1% yeast extract, and 0.4% K_2_HPO_4_.3H_2_O (pH 7.0). COS fermentation medium (%, w/v) contained 0.06% KH_2_PO_4_, 0.06% peptone, 1% COS, 0.1% yeast extract, 0.14% K_2_HPO_4_.3H_2_O, and 0.1% MgSO_4_.7H_2_O (pH 7.0).

### Screening of Chitosanase-Producing Strains

The soil samples were collected (5–10 cm deep) at Xisha Bay, Quanzhou, Fujian, China (N24°52′45″, E118°55′32″), and immediately diluted (10^–1^ to 10^–9^) with sterile water (30°C, 150 rpm). The bacterial suspensions (10^–2^ to 10^–6^) were spread over 0.45-μm Millipore membranes placed on a COS agar medium and incubated at 30°C for 5–7 days. Surviving colonies containing a specific clear zone were screened by primary screening for COSE-producing strains. The hydrolysis zone was measured based on the ratio (D/d) of transparent zone diameter (D) and colony diameter (d). To screen a strain with high COSE-producing potential, the isolated colonies were transferred to the previously described COS fermentation medium and cultured at 30°C for 72 h. The fermentation supernatants were collected to measure the COSE activity by using the DNS method. High COSE-producing potential strains were identified by morphology (optical microscope), biochemistry (gram staining, spore staining, and flagellar staining), and molecular biology [16S ribosomal RNA (rRNA)] methods. Gram staining was performed according to the method described by Claus (1992). Spore and flagellar staining were carried out by using procedures described by Mayfield and Inniss (1977). The genomic DNA of isolated high COSE-producing strain C4 was extracted using a Tiangen DNA kit (Tiangen Biotech, Beijing, China). PCR amplification of the 16S rRNA gene was carried out by using procedures described by Wei et al. (2021). Molecular phylogenetic analysis was performed by using MEGA7 software *via* the maximum likelihood method (Xue et al., 2020).

### Experimental Design and Optimization of Chitosanase Production

An investigation of the factors in the COS fermentation medium that affected the COSE activity was performed, with one factor checked at a time, including the carbon sources (1.0%, w/v, COS, maltose, starch, lactose, glucose, and sucrose), nitrogen sources [0.06%, w/v, peptone (NH_4_)_2_SO_4_, NH_4_NO_3_, KNO_3_, NaNO_3_, and NH_4_Cl], and trace elements (0.1%, w/v, NaCl, CuSO_4_, FeCl_2_, MgSO_4_.7H_2_O, MnSO_4_, CaCl_2_, and ZnSO_4_). The optimal COS, peptone, and MgSO_4_.7H_2_O concentrations were investigated at 0.5–2.0, 0.1–0.5, and 0.1–0.5%, respectively. The fermentation process parameters were investigated in a previously optimized medium. First, the COSE-producing strain was inoculated (1:100) into a 10-ml COS liquid medium and incubated at 30°C for 72 h by shaking at 150 rpm. The strain was then induced under a range of temperatures (25–37°C), inoculum sizes (1.0–5.0%), and liquid volumes (20–150 ml). According to the single-factor experimental results, a two-level P-B design (P-B) was first performed to assess the effects of five factors on COSE production: COS (g/L, X1), peptone (g/L, X2), MnSO_4_.7H_2_O (g/L, X4), liquid volume (L, X6), and inoculum size (%, v/v, X7). The steepest ascent experiment was performed to determine the optimal fermentation conditions based on the P-B experimental results. The RSM was carried out according to the P-B and steepest ascent results, and the influence of the three most significant variables was investigated: COS (g/L, X1), peptone (g/L, X2), and liquid volume (L, X3). Each variable was examined at low (−1), basal (0), and high (1) levels as follows: from 14.0 to 18.0 g/L (X1), 4.0 to 8.0 g/L (X2), and 30 to 40 L (X3).

### Purification of Chitosanase

The higher COSE-producing strain was inoculated (1:50) into a 150-ml optimized medium and cultured at 30°C for 72 h by shaking at 150 rpm. The crude enzyme was obtained by centrifugation at 6,000 rpm for 20 min. The crude enzyme was fractioned through precipitation with ammonium sulfate (NH_4_)_2_SO_4_ at 10, 20, 30, 40, 50, 60, 70, 80, 90, and 100% and was maintained at 4°C overnight. The protein concentration was measured according to the described method by Bradford (1976). The concentrated enzyme was loaded onto a Sephadex G-75 column (1.5 × 45 cm) equilibrated with 0.2-M acetic acid–sodium acetate buffer (HAc-NaAc, pH 5.6). The active fractions were pooled, and molecular weight was determined by the sodium dodecyl sulfate–polyacrylamide gel electrophoresis method (Sharma et al., 2020).

### Characterization of Chitosanase Enzyme

The effect of temperature on COSE activity was carried out by incubating at different temperatures (20–70°C) for 30 min. The thermal stability of COSE was determined by incubating at varying temperatures ranging from 30 to 50°C for different holding times (30–120 min). The effect of pH on COSE activity was carried out by incubating at different pH values [0.2-M HAc-NaAc buffer, pH 3.8–5.6, and 0.2-M phosphate buffer (PB), pH 6.0–7.6] for 30 min at 40°C. The pH stability of COSE was detected by incubating in different buffers of pH ranging from 3.8 to 7.6 for 15, 30, 45, 60, 75, and 90 min at 40°C. The purified enzyme was incubated and assayed in the presence of 20-mM CuSO_4_, MgSO_4_, CaCl_2_, ZnSO_4_, KCl, FeCl_3_, FeSO_4_, NaCl, and MnCl_2_ in 0.2-M PB (pH 7.2) at 40°C. The final concentration of metal ions in the reaction medium was 1 mmol/L. The abilities of the purified enzyme against water-soluble COS, 1% colloidal COS, powdered COS, powdered chitin, hydroxyethyl COS, and carboxymethyl chitin were detected by a reaction mixture containing 100-μl enzyme, 400-μl PB (0.2 M, pH 7.2), and 500-μl substrate (1% w/v). The results obtained from the hydrolysis of the substrate were used to calculate the enzyme kinetic parameters (*K*_*m*_ and V_*min*_) using the Lineweaver–Burk plot methods (Abbasi et al., 2020).

### Analysis of Chitosan Hydrolyzate

Chitosan (0.5 g) was dissolved in 0.5 ml of HAc (pH 5.6) to make a 1% colloidal solution. The reaction mixture of 10 ml of colloidal solution and 1 ml of crude enzyme solution was incubated at 30°C for 30 min. After centrifugation, the supernatant was obtained and used as the COS hydrolyzate. The COS hydrolyzate with different gradients was obtained by the processing methods mentioned earlier by adjusting the volume ratio of COS and crude enzyme solution to 5:1 and 1:1, respectively. COS hydrolyzates were spotted by preparative thin-layer chromatography (TLC) using silica gel plates and a solution of formaldehyde: methanol: 25% ammonia: water (5:10:1.5:1). A 20 mg/ml glucosamine hydrochloride solution as control was visualized on a TLC plate using a 0.1% ninhydrin solution. The hydrolysis ability of the COS degradation products was analyzed by high-performance liquid chromatography (HPLC). The supernatant of COS hydrolyzates obtained was filtered with a 0.22-μm membrane and further used for HPLC analyses with a PDA detector at 30°C. The mobile phase was a mixture of acetonitrile and water (v/v, 75/25), and the flow rate was 1 ml/min.

### Whole-Genome Sequencing and Assembly

The genomic DNA of isolated high COSE-producing strain C4 was extracted using a Tiangen DNA kit (Tiangen Biotech, Beijing, China), and the integrity of DNA was checked with agarose gel electrophoresis. The purity and concentration of DNA were analyzed by using a NanoDrop 2000 spectrophotometer (Thermo Scientific, United States). To ensure adequate read lengths of the genome assembly of *Mitsuaria* sp. C4, next-generation sequencing on the Illumina HiSeq X Ten platform and third-generation sequencing on the PacBio SEQUEL platform were applied for whole-genome sequencing. Approximately 1.70- and 1.74-Gb clean data were generated by PacBio and Illumina, respectively. The data were corrected, trimmed, and assembled using CANU (version 1.6) with the parameters corOutCoverage = 80 and corMinCoverage = 0, then further polished by the Pilon programs with default parameters (Xue et al., 2020). The completeness of the C4 genome was assessed with BUSCO (Simão et al., 2015).

### Function Annotation

Coding genes of the C4 genome were predicted with the GeneMark program (version 2.5).^1^ Non-coding RNA and small RNAs were predicted by alignment to the Rfam and miRNA databases using tRNAsan-SE and BLASTN, respectively (Liu et al., 2020). Functional annotation of the protein-coding genes in the EggNOG, Gene Ontology (GO), Clusters of Orthologous Genes (COG), Kyoto Encyclopedia of Genes and Genomes (KEGG), and carbohydrate-active enzyme (CAZy) databases was performed using BLASTP software (Lombard et al., 2014; Xue et al., 2020). CAZy can be classified into five classes, including glycoside decomposing enzymes (GHs), glycosyltransferases (GTs), polysaccharide lyases (PLs), carbohydrate-binding modules (CBMs), carbohydrate esterases (CEs), and oxidoreductases (AAs) (Park et al., 2018).

## Results and Discussion

### Isolation, Screening, and Identification of Chitosanase-Producing Microorganisms

**FIGURE 1:**
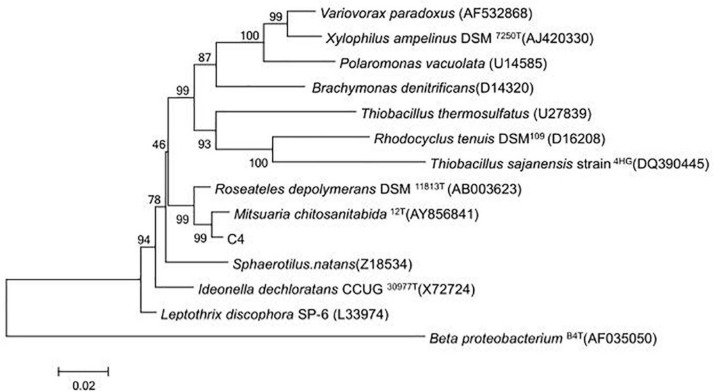
Evolutionary tree based on 16S rDNA from strain C4 and other closely related species.

In total, 19 isolated bacterial strains produced a clear zone and underwent COSE hydrolysis on COS agar medium, and six strains (C1, C4, C5, C18, C43, and C82) had high COSE activity. Strain C4 exhibited the highest COSE activity (6.235 U/ml) and D/d ratio (D/d = 4.10 ± 0.12) and was selected as the most promising COSE-producing bacterium (Table 1). Strain C4 is a Gram-negative, non-spore, capsule, and long flagellated aerobic bacterium according to the morphology and biochemistry analyses (Supplementary Figure 1). A phylogenetic tree analysis showed that the isolated C4 had a high similarity (99.0%) to *M. chitosanitabida* by BLASTing 16S rRNA (approximately 1.5 kb) (Figure 1). Studies have shown that the standard value of an average nucleotide identification (ANI) for species classification in the prokaryotic classification system mainly ranged from 90.0 to 96.0% (Liu et al., 2013). By comparing the ANI value from C4 with the other six kinds of *Mitsuaria* sp., we found that the ANI value is below 92.0% with a wide range from 84.7 to 91.7% (Supplementary Table 1). The result indicated that strain C4 might belong to the *Mitsuaria* sp. and was close to the model organism *M. chitosanitabida* with an 84.73% true ortholog value. Therefore, *Mitsuaria* sp. C4 was used for subsequent studies.

**TABLE 1:** Comparison of transparent zone and enzyme activity in chitosanase-producing strains.

**Sample**	**Transparent zone diameter (D, cm)**	**Colony diameter (d, cm)**	**D/d**	**Chitosanase activity (U/ml)**
C1	1.61	0.68	2.37 ± 0.11	3.844
C2	0.45	0.21	2.14 ± 0.08	1.612
C3	0.51	0.23	2.22 ± 0.15	1.452
C4	2.01	0.49	4.10 ± 0.12	6.235
C5	1.18	0.35	3.37 ± 0.05	3.010
C6	0.76	0.31	2.45 ± 0.07	2.373
C7	0.51	0.3	1.70 ± 0.09	1.299
C9	0.62	0.29	2.14 ± 0.10	1.901
C10	0.98	0.35	2.80 ± 0.12	2.447
C14	1.25	0.61	2.05 ± 0.13	1.710
C15	1.05	0.4	2.63 ± 0.06	2.091
C18	0.97	0.38	2.55 ± 0.18	2.566
C26	0.88	0.45	2.00 ± 0.05	1.940
C28	0.85	0.33	2.58 ± 0.15	2.543
C43	1.13	0.36	3.14 ± 0.11	2.632
C77	1.1	0.52	2.12 ± 0.12	2.296
C78	0.98	0.47	2.09 ± 0.09	2.195
C81	0.67	0.31	2.16 ± 0.07	2.070
C82	1.24	0.33	3.76 ± 0.12	3.899

### Preliminary Optimization of the Medium Compositions and Process Parameters by Single-Factor Experiments

**FIGURE 2:**
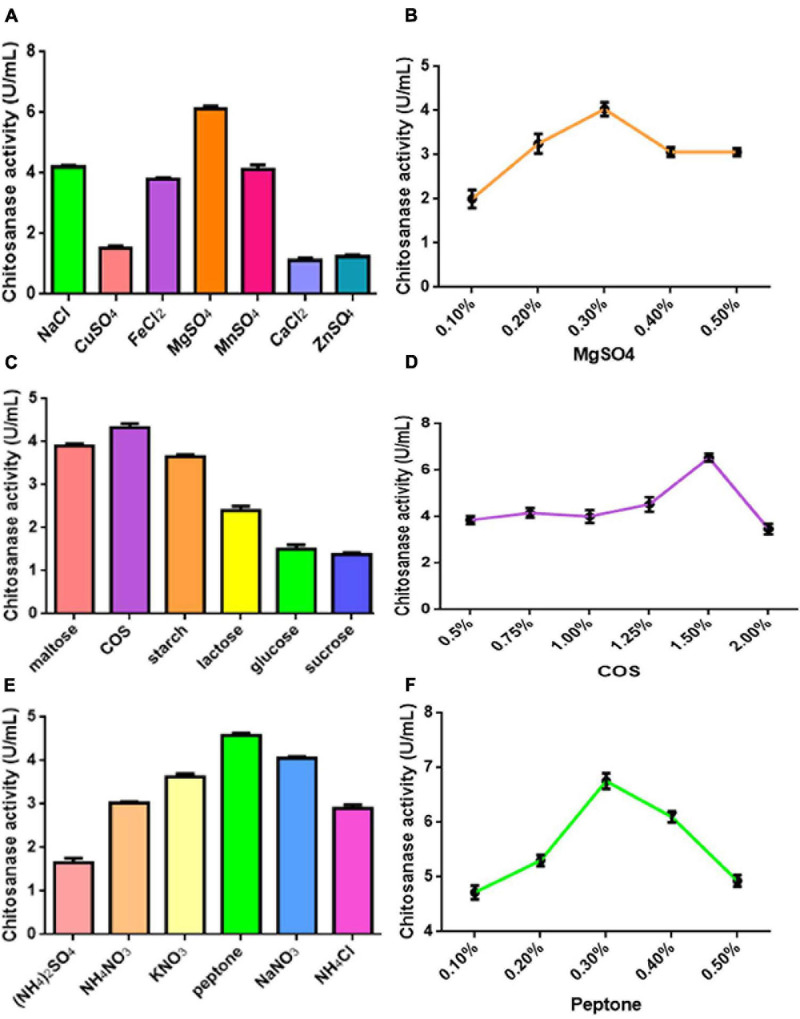
**(A)** Effect of different kinds of metal ions on COSE activity. **(B)** Effect of different concentrations of MgSO_4_ on COSE activity. **(C)** Effect of carbon source on COSE activity. **(D)** Effect of different concentrations of COS on COSE activity. **(E)** Effect of nitrogen sources on COSE activity. **(F)** Effect of different concentrations of peptone on COSE activity.

Various COS fermentation medium designs and process parameters were optimized for the highest COSE activity using the one factor at a time method. The optimized medium compositions for C4 fermentation were as follows (w/v): 1.5% COS, 0.3% peptone, and 0.3% MnSO_4_.7H_2_O. Maximum COSE activity (6.837 U/ml) was detected after 72 h of incubation at 30°C with an inoculum size of 4% in a 100-ml optimized COS fermentation medium (Figure 2). These results can be used as the research basis for the optimization of fermentation conditions in the following parts.

### Optimization of Fermentation Conditions by Response Surface Methodology

**FIGURE 3:**
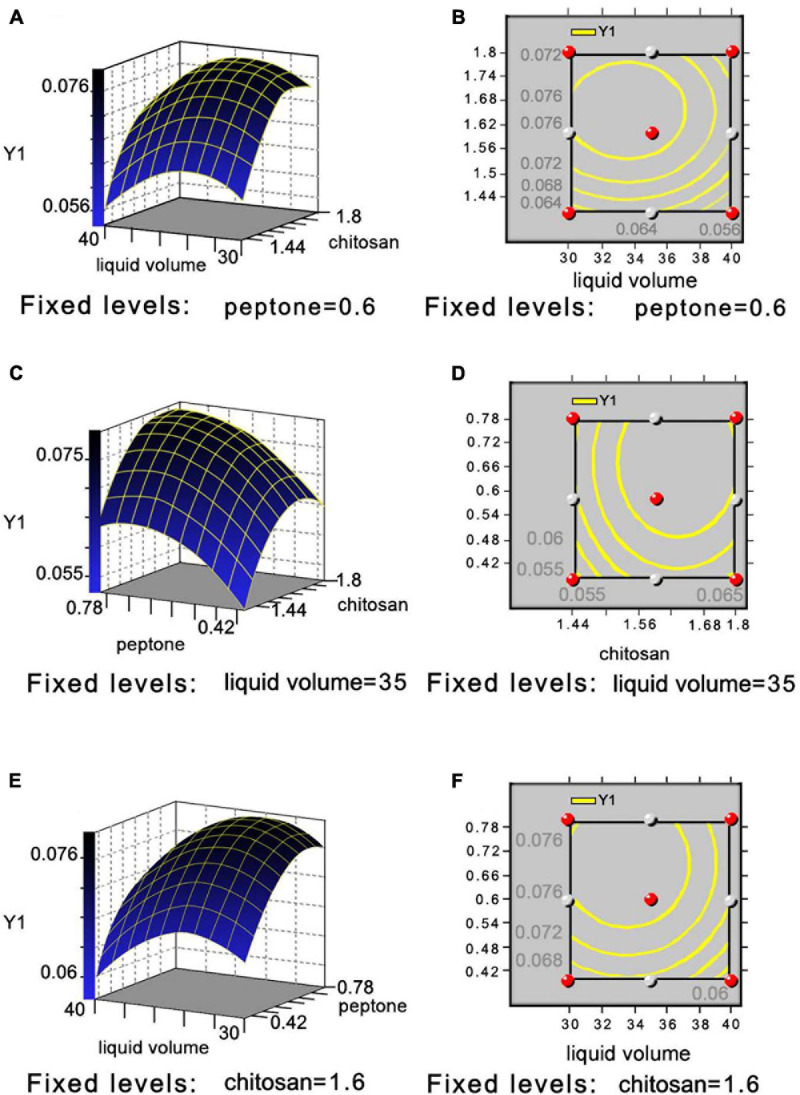
Three-dimensional response surface and two-dimensional contour plot showing effects between liquid volume and COS **(A,B)**, peptone and COS **(C,D)**, and liquid volume and peptone **(E,F)** on COSE activity.

The *P*-values of the COS, peptone, and liquid volume (Pr > | t|) for evaluating the effects of the three medium components and two process conditions were <0.01 by the P-B design, indicating its significant effects on COSE production (Supplementary Tables 2, 3). Run 3 showed the highest COSE (7.365 U/ml) and was used as the central point of the RSM design according to the results of the steepest ascent design (Supplementary Table 4). The mean value of the predicted COSE activity was calculated using the Box–Behnken method (Khajeh, 2012). Four experimental runs (runs 7, 13, 14, and 15) showed high COSE production levels based on the enzyme activity results at 7.515, 7.666, 7.515, and 7.591 U/ml, respectively (Supplementary Tables 5, 6). The optimum COSE activity (7.666 U/ml) was obtained in run 13, and the optimized level was selected. The multiple regression analysis showed that the *R*^2^ and adjusted *R*^2^ values of the model were 0.9611 and 0.8910, respectively, which indicated satisfactory compatibility between the theoretical and experimental results of COSE production by the model equation (Supplementary Table 7). The results from the parameter estimation by the model equation showed that X1 (*P* = 0.004 < 0.01), X2 (*P* = 0.004 < 0.01), X1 × X1 (*P* = 0.0018 < 0.01), and X3 × X3 (*P* = 0.0096 < 0.01) had a very significant effect on COSE production, and the factors with the greatest degree of influence on the equation were COS > peptone > liquid volume (Supplementary Table 8). The lack of fit (*P* = 0.0682 > 0.05) was insignificant, indicating that the equation fit the test well. The optimal values of the three independent variables were carried out to illustrate the influence on the maximum COSE activity by drawing the three-dimensional response surface and two-dimensional contour map. The COSE activity varied significantly with the liquid volume (X3) and concentrations of COS (X1) and peptone (X2). When the liquid volume was 35 ml, the COSE activity significantly increased first as the COS concentration increased, and then it decreased, and the peptone concentration was fixed at its optimal level (w/v, 0.6%), as shown in Figures 3A,B. The COSE activity increased rapidly as the percentage of COS increased from 1.44 to 1.64%; it also increased as the percentage of peptone increased from 0.42 to 0.65% but decreased as the values of COS and peptone increased from 1.64 to 1.80% and 0.65 to 0.78% (Figures 3C,D). The results indicated that the percentage of COS and peptone at a fixed liquid volume (35 ml) was positively correlated with increasing COSE activity. The relationship between the liquid volume and peptone concentration on the COSE activity was significant (Figures 3E,F) when the COS percentage was 1.64%. Similarly, liquid volume and peptone concentration also had significant interaction, suggesting enhancement of COSE activity. In summary, mathematical models indicated that the liquid volume and the concentrations of COS and peptone had a significant effect on COSE activity. The highest COSE activity (8.344 U/ml) was obtained at the following critical values: COS, 1.64%; peptone, 0.65%; liquid volume, 35 ml; temperatures, 30°C; inoculum size, 1%; and incubation time, 72 h. The highest COSE activity was 1.34-fold higher than that of the preoptimized conditions (6.235 U/ml) and 1.22-fold higher than that of preliminary optimization by single-factor experiments (6.835 U/ml). This optimized scheme can be used to prepare COSE on a large scale at a low cost. These results provide a basis and reference model for maximizing the industrial production and application of COSE.

### Isolation and Purification

**FIGURE 4:**
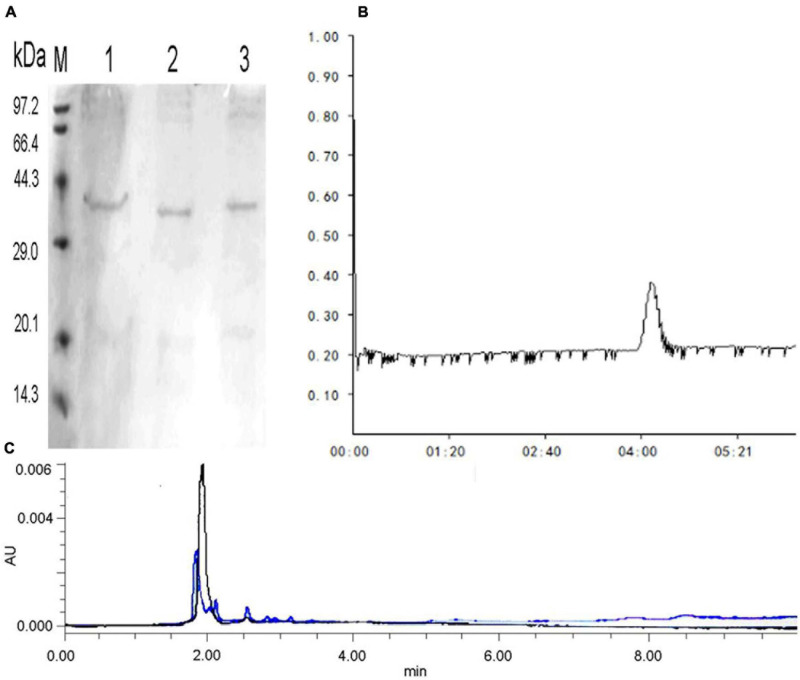
**(A)** SDS-PAGE electrophoresis of COSE protein obtained by Sephadex G-75 chromatography. Lane M: standard molecular weight (kDa); Lane 1: 20 mg/ml protein; Lanes 2–3: 10 mg/ml protein. **(B)** Sephadex G-75 of COSE. **(C)** Analysis of COS hydrolyzates by HPLC. Blue peaks indicate mixed standard of COS degradation products. Black peaks represent COS degradation products by purified COSE from C4 strain.

After precipitation with ammonium sulfate, the specific activity of the purified enzyme reached 14.52 U/mg protein, with a 38.46% recovery rate and 4.79-fold purification by Sephadex G-75 (Supplementary Table 9). The relative molecular mass of the purified enzyme was calculated by sodium dodecyl sulfate–polyacrylamide gel electrophoresis, which presented only one protein band at approximately 34 kDa (Figures 4A,B). The molecular mass of C4 COSE (34 kDa) was approximately the same as that of other *Mitsuaria* sp. and *Bacillus* sp., including *Mitsuaria* sp. 141-2 (31 kDa), *M. chitosanitabida* 3001 (34 kDa), *Bacillus cereus* 6E1 (36 kDa), and *Bacillus* sp. X-b (35 kDa) (Helistö et al., 2001; Wang et al., 2001; Yun et al., 2006). The molecular mass of C4 COSE (34 kDa) was completely different from that of most *Bacillus* sp., such as *B. cereus* TKU022 (44 kDa), *Bacillus* sp. MET1299 (52 kDa), *Bacillus* sp. 739 (46 kDa), *Bacillus* sp. DAU101 (27 kDa), *Bacillus* sp. P16 (45 kDa), and *Bacillus* sp. BG-11 (41 kDa) (Aktuganov et al., 2003; Lee et al., 2006; Jiang et al., 2012). The purified COSE mentioned earlier had stable enzyme activity, high specific activity, and high recovery rate. The relative molecular weight of the purified enzyme was slightly smaller than that of most previous enzyme proteins obtained by researchers from other microorganisms. The COSE protein can be successfully obtained by the isolation and purification scheme established earlier.

### Effect of pH, Temperature, and Metal Ions

The pH activity profiles of C4 COSE exhibited maximum activity at pH 7.2, which was similar to that of *Penicillium janthinellum* D4 (pH 7–9) (Wu and Zhang, 2019). However, the C4 COSE activity was obviously different from that of most fungal (*Aspergillus* sp., 3.5–6.5) or bacterial COSE (*Bacillus* sp., pH 3.0–10.0; *Pseudomonas* sp., pH 5.0–8.0; and *Streptomyces* sp., 3.0–8.0) (Lee et al., 2006; Ando et al., 2008; Jiang et al., 2012), which displayed maximum activity at pH 7.2 (Supplementary Figure 2). Studies showed that when the pH value is higher than 6.5, the enzyme activity will significantly decrease (Zhou et al., 2010; Nguyen et al., 2014). The C4 COSE pH stability profiles were determined at pH 7.2 at different times (15–90 min), and relatively stable profiles were observed at pH 7.2 at 30 min (Supplementary Figure 2). When the temperature was higher than 40°C, the decreasing trend of enzyme activity was stable. The optimum temperature and thermal stability for C4 COSE activity was 40°C. More than 70% activity was retained for 30 min, which was similar to that of *B. cereus* TKU030 (40°C), but a significantly different result was demonstrated by closely related strains, such as *Mitsuaria* sp. 141-2 (30°C) and *M. chitosanitabida* 3001 (50°C). The thermal stability of C4 COSE was lower than that of other bacterial strains, such as *Bacillus mycoides* (50°C), *Bacillus subtilis* PT5 (47°C), *Bacillus amyloliquefaciens* (50°C), and *Paenibacillus mucilaginosus* TKU032 (70°C) (Liang et al., 2012; Su et al., 2017; Qin et al., 2018b; Doan et al., 2019). To further characterize C4 COSE, the effect of various metal ions on COSE activity was examined. The COSE activity was 70, 77, 50, 80, and 55% reduced in the presence of 1-mM Ca^2+^, K^+^, Fe^2+^, Mn^2+^, and Na^+^, respectively. Interestingly, the COSE activity was significantly suppressed by 1-mM Cu^2+^ and Fe^3+^, resulting in 100 and 95% inhibition, respectively (Supplementary Figure 2). *P. janthinellum* D4 COSE activity was incubated with 5-mM Cu^2+^, resulting in 95% inhibition. We speculated that Cu^2+^ could make the tertiary structure of the protein more unstable at low concentrations, thereby inhibiting the stability of the enzyme. The pH activity curve of C4 COSE shows the maximum activity and good thermal stability at pH 7.2. These characteristics are favorable for its application. The alkaline enzymatic hydrolysis medium has lower requirements on the reactor than the acidic medium. Cu2^+^ and Fe^3+^ will significantly inhibit the activity of COSE; it is necessary to pay attention to the container and tools in contact with C4 COSE instead of using metal and try to use an enamel kettle.

### Effect of Substrate Specificity and Kinetic Constant Determination

The purified COSE activity toward water-soluble COS, 1% colloidal COS, powdered COS, powdered chitin, hydroxyethyl COS, and carboxymethyl chitin substrates was detected. The enzyme showed high activity toward powdered COS and 1% colloidal COS and carboxymethyl chitin (Supplementary Figure 2). This result indicates that C4 COSE has a good degradation effect on COS and exhibits substrate specificity. The *K*_*m*_ and V_*min*_ of the COSE toward COS were calculated as 2.449 g/L and 0.042 g/min/L, respectively (Supplementary Figure 3 and Supplementary Table 10).

### Analysis of the Chitosan Hydrolyzates

Chitooligomer formation was observed in the COS hydrolyzates with different ratios of COS and crude enzyme solution (v/v, 10:1 or 5:1 or 1:1) by TLC analysis (Supplementary Figure 3). HPLC analysis revealed that the produced purified COSE could produce a mixture of oligosaccharides other than monosaccharides after enzymatic hydrolysis (Figure 4C). The results indicated that purified COSE from C4 might be an endonuclease based on the reaction mentioned earlier. Generating oligosaccharides by COSE hydrolysis is an attractive synthesis method (Yun et al., 2006). However, COS hydrolyzates are difficult to use on a large scale due to their high production costs and low yield (Lee et al., 2006). Several attempts to produce chitooligomers by microbial COSE have been conducted in recent years. For example, COSE has been produced by *Purpureocillium lilacinum* CFRNT12, which is an endonuclease that forms only chitooligomers (Nidheesh et al., 2015). COSE has been produced by *Aspergillus* sp. Y2K, which hydrolyzes COS to produce oligochitosan (Cheng and Li, 2010). COSE is produced by *P. janthinellum* D4 hydrolysis of COS and obtains a good yield of chitooligomers (DP, 3–9) (Ando et al., 2008). The COSE-producing strain obtained can ferment in mild conditions with high enzyme production efficiency and low cost. These results indicated that C4, as a potential new strain, could be used to produce COSE for the hydrolysis of COS into chitooligomers. The obtained COSE stability is good, for which enzymatic hydrolysis reaction system is easy to control, and the production equipment requirements are rather low. The yield of chitooligomers obtained from COSE enzymatic hydrolysis is high, and the products can be used as raw materials for producing healthy, functional foods.

### Whole-Genome Sequencing, Assembly, and Annotation

**FIGURE 5:**
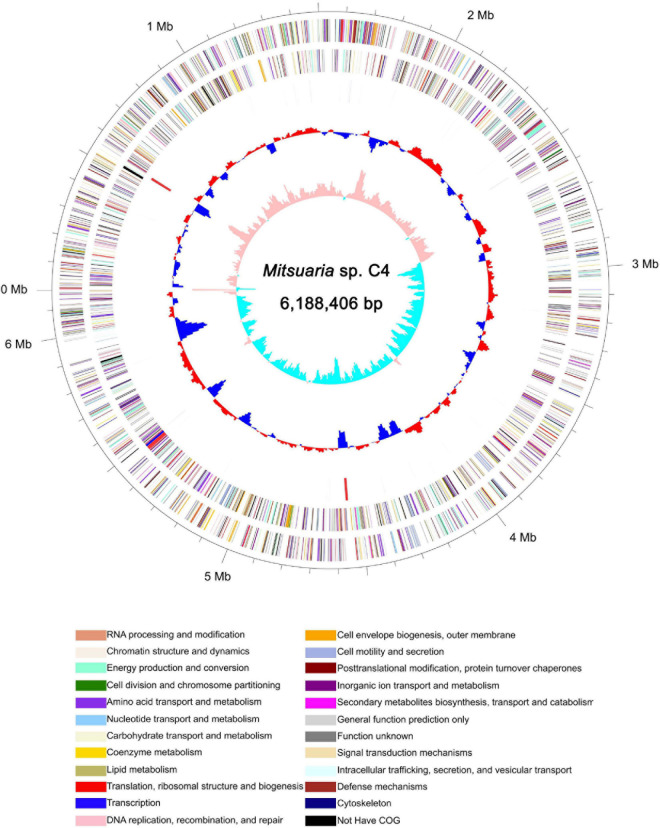
Schematic of complete *Mitsuaria* sp. C4 genome. Order of circles from outermost to innermost is as follows: genome size (first circle), CDS on forward and reverse strands (second and third circles), COG categories, rRNA and tRNA (fourth circle); GC content (fifth circle), and GC skew (sixth circle).

To understand the genomic characteristics of C4, whole-genome sequencing was performed. Genome assembly demonstrated that the C4 genome size was 6.18 Mb with a GC content of 68.40% and 5,268 protein-coding genes (Figure 5, Supplementary Figure 3 and Supplementary Tables 11, 12), which was obviously different from that of other *Mitsuaria* sp., such as *Mitsuaria* sp. 7, *Mitsuaria* sp. BK037, *Mitsuaria* sp. BK041, *Mitsuaria* sp. BK045, *Mitsuaria* sp. HWN-4, *Mitsuaria* sp. PDC51, *Mitsuaria* sp. TWR114, *Mitsuaria* sp. WAJ17, *M. chitosanitabida*, and *Micromonospora noduli* (Supplementary Table 13; Yun et al., 2005; Rong et al., 2012; Sisinthy and Gundlapally, 2020). Approximately 97.97% of orthologs were included in the assembled sequences, indicating a high degree of completeness for the genome assembly. Non-coding RNAs in C4 were annotated by searching the Rfam database, including 62 transfer RNAs, 12 rRNAs, and 3 soluble RNAs (Supplementary Table 14). Repetitive sequences of the C4 genome make up 1.97% of the whole genome (Supplementary Table 15). We functionally annotated 4,460, 1,701, 4,209, and 1,881 genes to EggNOG, GO, COG, and KEGG, respectively, obtaining 4,553 genes (86.42% of the total) with at least one hit in public databases (Supplementary Table 16). A GO analysis showed that a large number of genes were related to cellular processes (1,087 genes), metabolic processes (1,056 genes), cells (1,176 genes), and catalytic activity (824 genes) (Supplementary Figure 4). The COG annotation results showed that the transport and metabolism functions of amino acids and carbohydrates accounted for 7.89% (332 genes) and 7.18% (204 genes) of the COG categories (4,209 genes), respectively (Supplementary Figure 4). The COG terms of the major facilitator superfamily were the most abundant in carbohydrate transport and metabolism, followed by amino acid ABC-type carriers, polysaccharide deacetylases, and transporters. Studies have reported that the major facilitator superfamily, which is a secondary active transporter, catalyzes substrate transport, including carbohydrates, amino acids, and nucleosides (Rossi et al., 2002). A total of 308 and 40 genes were related to carbohydrate metabolism (16.37%) and sucrose and starch metabolism (2.13%) of the pathway classes by the KEGG analysis, respectively (Supplementary Figure 4). For carbohydrate metabolism, COSE (EC 3.2.1.132, orf_531) was identified as a specific enzyme that plays an essential function in the hydrolytic degradation of COS. Studies have shown that cellulase, as a non-specific enzyme, has COS hydrolase active ingredients. For example, novel COSE from *Streptomyces griseus* HUT 6037 can hydrolyze deacetylated COS and carboxymethylcellulose with transglycosylation activity (Tanabe et al., 2003). COSE from *Streptomyces olivaceus* strain FXJ showed catalytic activity in the hydrolysis of COS and carboxymethylcellulose (Yue et al., 2016). C4 generates a large amount of energy when it metabolizes and degrades COS products, which can be used for the metabolism and synthesis of other carbohydrates and amino acids. A total of 181 genes were identified in energy pathways, and they accounted for 9.62% of the KO terms, including oxidative phosphorylation (68 genes) and carbon fixation (61 genes). D-glucose can be converted to D-glucose 6-phosphate by phosphorylation, which is involved in pentose phosphorylation and carbohydrate metabolism (Moonira et al., 2020). We speculated that the enhancement of oxidative phosphorylation makes it possible for the bacteria to use available carbohydrates in the degradation process. There are a large number of genes encoding enzymes related to carbohydrate metabolism in the C4 genome sequence, and subsequent studies can clone these genes with excellent characteristics through genome mining, which can be used for the degradation reaction of polysaccharides.

### CAZy Families Involved in Carbohydrate Degradation

We identified 211 carbohydrate enzymes, including 35 CBMs (16.59%), 51 CEs (24.17%), 60 GHs (28.44%), 44 GTs (20.85%), 5 PLs (2.37%), and 16 AAs (7.58%) (Supplementary Figure 4). Analysis of the CAZy families showed that the GH family was divided into 18 subcategories, including 2 GH1, 2 GH3, 4 GH16, and 4 GH18. Enzymes of the GH family are involved in the cleavage of polymeric substrates (Fushinobu et al., 2013). COSEs belong to GHs-8 and include 46 and 80 families mainly derived from bacteria, of which the GHs-46 family mainly corresponds to *Bacillus*, *Streptomyces*, *Nocardioides*, *Burkholderia*, and *Chlorella* virus, whereas the GHs-80 family primarily corresponds to *Matsuebacter chitosanotabidus* and *Sphingobacterium multivorum* (Tremblay et al., 2000). COSE CsnA from *Streptomyces coelicolor* A3(2) had an antibacterial effect on COS and acted as a potential protective enzyme (Ghinet et al., 2010). The GHs-46 subclass III COSE from *Bacillus circulans* MH-K1 effectively inhibited the growth of *Rhizopus* and *Mucor* (Minoru et al., 2006). Interestingly, we identified a GHs-46 COSE and four GHs-18 chitinase genes that play an important role in the preparation of partially acetylated COS. The metabolic potential for oligosaccharide-degrading enzymes (GH1, GH3) was found in C4, and this result is inconsistent with a report on the reconstructed genomes that did not identify the GH2, GH42, and GH43 families (Weimann et al., 2013). In addition, CBM32 modules have been confirmed to enhance catalytic activity by binding to COS oligosaccharides (GlcN)_2__–__6_. CBM32 at the C-terminus of a COSE from *Paenibacillus* sp. IK-5 was first identified and specifically bound COS oligosaccharides (Shinya et al., 2016). In total, 35 CBMs were detected in the C4 genome, and they may play roles in the COS degradation process. We found that the number or member type of CAZy families based on the C4 genome differed significantly from that of other genomes. For example, five PL members were identified in C4 but not in *Bacillus thermoamylovorans* (Krawczyk et al., 2015). A total of 211 carbohydrate enzymes were predicted in the C4 genome, which is higher than that of *B. thermoamylovorans* (96). The GH member number of C4 (60) is lower than that of *Paenibacillus lautus* strain BHU3 (143) (Yadav and Dubey, 2018), which is likely because of the specificities of the species and the different target products of biodegradation. The diversity of the CAZys families indicates that C4 could be a good resource for enhancing carbohydrate degradation and a useful tool for oligosaccharide metabolism.

### Secondary Structure Prediction and Three-Dimensional Modeling of COSE

**FIGURE 6:**
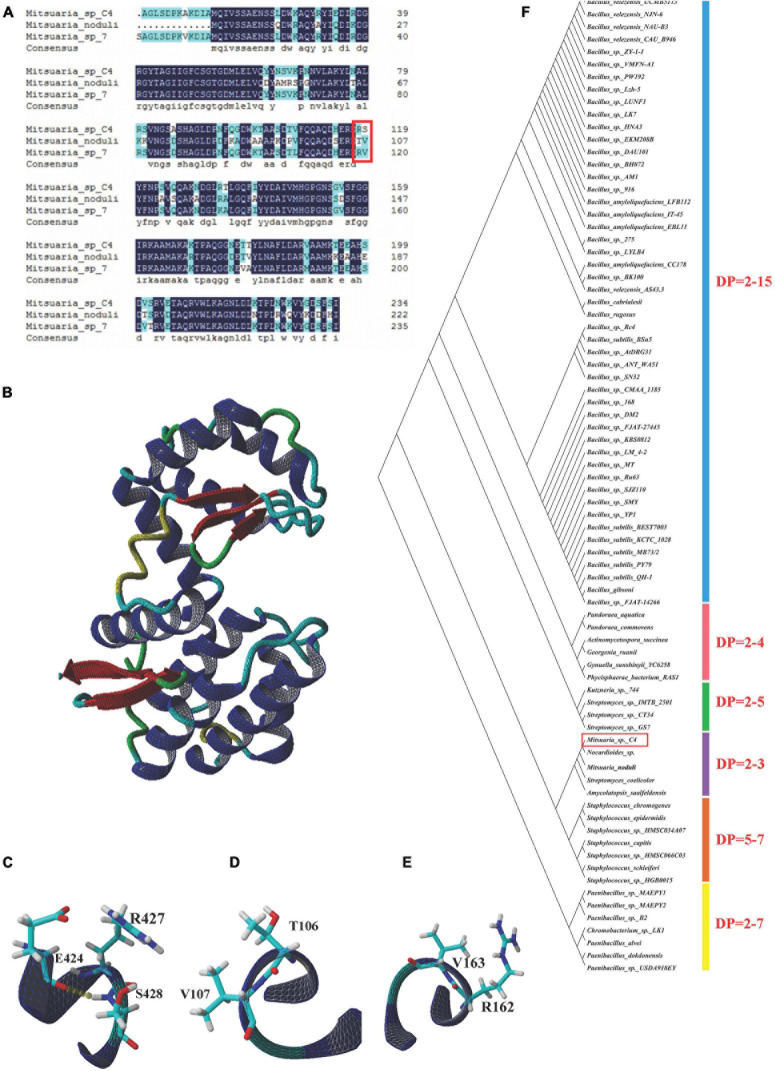
Bioinformatic analysis of COSE. **(A)** Multiple amino acid sequence alignment of COSE belonging to family GHs-46 in *Mitsuaria* sp. C4, *Mitsuaria* sp. 7, and *M. noduli*. Typical catalytic sites were emphasized with red square. **(B)** Three-dimensional structures of COSE in *Streptomyces* sp. N174. **(C)** Region of COSE with Arg and Ser sites in *Mitsuaria* sp. C4. **(D)** Region of COSE with Arg and Val sites in *M. noduli*. **(E)** Region of COSE with Thr and Val sites in *Mitsuaria* sp. 7. **(F)** Phylogenetic analysis of GHs-46 family COSEs. Phylogenetic divergence was analyzed using MEGA 7.0 software.

We found that COSEs obtained from *Mitsuaria* sp. belong to GHs-46 and GHs-80 families, of which the GHs-46 family corresponds to *Mitsuaria* sp. 7, C4 and *M. noduli* based on the domain identification. The COSE sequence homology of the three species discussed earlier is highly conserved (reaching more than 90.0%) (Figure 6A). Three-dimensional structure of COSE from *Streptomyces* sp. N174 was constructed as a template by SWISS-MODEL server, and the structural model of *Mitsuaria* sp. 7, C4 and *M. noduli* were analyzed by YASARA software (Figure 6B). In amino acid substitution models, there are some different sites at Arg_162_–Val_163_ (*M. noduli*). The differences of Thr_10__6_–Val_107_ (*Mitsuaria* sp. 7) and Arg_427_–Ser_428_ (C4) residues of COSE lead to differences in their secondary structure (Figures 6C–E). Interestingly, the COSE obtained from C4 exhibited a large difference distributing in the Arg_427_–Ser_428_ compared with the other two strains, which produced a new hydrogen bond. The Arg_427_–Ser_428_ mutation of COSE in C4 with the formation of a new hydrogen bond may influence COSE activity and the charge relay system. The formation of new hydrogen bonds at Arg_427_–Ser_428_ by COSE obtained from C4 may be beneficial to the stability of COSE. The establishment of protein model of C4 can be used to achieve the targeted modification of the enzyme protein through point mutation. Phylogenetic analysis of the GHs-46 family COSE revealed that the GHs-46 family COSEs clustered with different DP based on their cleavage specificity (Figure 6F). The novel COSE obtained from C4 was clustered into the DP = two to three groups, which can perform catalysis in a similar manner to produce (GlcN)_2_ and (GlcN)_3_.

## Conclusion

In this study, the isolation, purification, and characterization of a novel endo-type COSE from a newly isolated C4 are reported. By comparing the ANI value from C4 and other six kinds of *Mitsuaria* sp., we found that strain C4 may belong to the *Mitsuaria* sp. and was close to the model organism *M. chitosanitabida* with an 84.73% true ortholog value. The highest COSE activity (8.344 U/ml) was achieved by optimization, and it was 1.34-fold higher than that obtained under preoptimized conditions (6.235 U/ml). Whole-genome sequencing and functional annotation indicated that the C4 strain could be a good resource for enhancing carbohydrate degradation and a useful tool for oligosaccharide production. These results provide a basis and reference model for maximizing the industrial production and application of COSE.

## Data Availability Statement

Whole-genome sequencing data of Mitsuaria sp. C4 were deposited in the BIG Sub system under BioProject accession number PRJCA004345. Datasets related to this article can be found at http://dx.doi.org/10.17632/rsg7c6665m.1.

## Author Contributions

YC and TX designed and coordinated the entire project and participated in manuscript writing and revision. CC, DC, and XZ performed the collection and processing of samples. JC, WH, CL, and HC performed the analyses of genome annotation. All authors read and approved the final manuscript.

## Conflict of Interest

The authors declare that the research was conducted in the absence of any commercial or financial relationships that could be construed as a potential conflict of interest.

## Publisher’s Note

All claims expressed in this article are solely those of the authors and do not necessarily represent those of their affiliated organizations, or those of the publisher, the editors and the reviewers. Any product that may be evaluated in this article, or claim that may be made by its manufacturer, is not guaranteed or endorsed by the publisher.

## Footnotes

1
http://topaz.gatech.edu/GeneMark/
