# Supplementary material for: Chitosan Oligosaccharide Production Potential of Mitsuaria sp. C4 and Its Whole-Genome Sequencing
Source: Front Microbiol. 2021 Aug 5;12:695571. doi: 10.3389/fmicb.2021.695571 (PMC8374441; doi:10.3389/fmicb.2021.695571)
Supplement: Supplementary file 1 [file Table_1.DOCX]

Supplementary Supplementary Tables

Supplementary Supplementary Table S1. Compare the ANI from six kinds of *Mitsuaria sp.* and *Mitsuaria sp.*C4.

| GenBank assembly accession | trueOrthoANI(%) |
| --- | --- |
| mit-GCA_000285635 | 90.2496 |
| mit-GCA_001598255.1 | 84.7326 |
| mit-GCA_001653795 | 90.0224 |
| mit-GCA_002205125 | 91.7253 |
| mit-GCA_002761755 | 86.6169 |
| mit-GCA_900113225 | 86.8549 |

Supplementary Table S2. Exprimental design and respose of Plackett-Burman（N=12）.

| Run | X1 | X2 | X3 | X4 | X5 | X6 | X7 | X8 | Chitosanase activity  （Y,（U/mL） |
| --- | --- | --- | --- | --- | --- | --- | --- | --- | --- |
| 1 | 1 | -1 | 1 | -1 | -1 | -1 | 1 | 1 | 3.748 |
| 2 | 1 | 1 | -1 | 1 | -1 | -1 | -1 | 1 | 4.351 |
| 3 | -1 | 1 | 1 | -1 | 1 | -1 | -1 | -1 | 3.899 |
| 4 | 1 | -1 | 1 | 1 | -1 | 1 | -1 | -1 | 3.899 |
| 5 | 1 | 1 | -1 | 1 | 1 | -1 | 1 | -1 | 4.276 |
| 6 | 1 | 1 | 1 | -1 | 1 | 1 | -1 | 1 | 3.974 |
| 7 | -1 | 1 | 1 | 1 | -1 | 1 | 1 | -1 | 3.824 |
| 8 | -1 | -1 | 1 | 1 | 1 | -1 | 1 | 1 | 3.748 |
| 9 | -1 | -1 | -1 | 1 | 1 | 1 | -1 | 1 | 3.598 |
| 10 | 1 | -1 | -1 | -1 | 1 | 1 | 1 | -1 | 3.748 |
| 11 | -1 | 1 | -1 | -1 | -1 | 1 | 1 | 1 | 3.522 |
| 12 | -1 | -1 | -1 | -1 | -1 | -1 | -1 | -1 | 3.899 |

Supplementary Table S3. Factor levels and significance of Plackett-Burman design

| Coding | Factor | Level | | t Value | Pr>\|t\| | Significant |
| --- | --- | --- | --- | --- | --- | --- |
|  |  | -1 | 1 |  |  |  |
| X1 | COS（g/L） | 10 | 15 | 4.08368 | 0.0265 | 1 |
| X2 | peptone（g/L） | 1 | 3 | 3.27020 | 0.0468 | 2 |
| X4 | MnSO4•7H2O（g/L） | 1 | 3 | 2.45671 | 0.0911 | 4 |
| X6 | liquid volume（L） | 0.05 | 0.1 | -3.67694 | 0.0348 | 3 |
| X7 | inoculum size（%，v/v） | 1 | 3 | -2.04455 | 0.1335 | 5 |

Supplementary Table S4. Experimental design and results of steepest ascent

| Run | COS（g/L） | peptone（g/L） | liquid volume（mL） | Y/(U/mL) |
| --- | --- | --- | --- | --- |
| 0 | 10 | 3.0 | 50 | 4.577 |
| 1 | 12 | 4.0 | 45 | 5.481 |
| 2 | 14 | 5.0 | 40 | 6.461 |
| 3 | 16 | 6.0 | 35 | 7.365 |
| 4 | 18 | 7.0 | 30 | 6.837 |
| 5 | 20 | 8.0 | 25 | 6.536 |

Supplementary Table S5. Design of Box-Behnken experiment.

| Level | Fator | | |
| --- | --- | --- | --- |
|  | X1(COS, g/L） | X2（peptone, g/L） | X3（liquid volume） |
| -1 | 14.0 | 4.0 | 30 |
| 0 | 16.0 | 6.0 | 35 |
| 1 | 18.0 | 8.0 | 40 |

Supplementary Table S6. Design and result of Box-Behnken experiment.

| Run | COS（g/L） | peptone（g/L） | liquid volume（mL） | Y/(U/mL) |
| --- | --- | --- | --- | --- |
| 1 | -1 | -1 | 0 | 5.858 |
| 2 | -1 | 1 | 0 | 6.611 |
| 3 | 1 | -1 | 0 | 6.310 |
| 4 | 1 | 1 | 0 | 7.063 |
| 5 | 0 | -1 | -1 | 6.461 |
| 6 | 0 | -1 | 1 | 6.084 |
| 7 | 0 | 1 | -1 | 7.515 |
| 8 | 0 | 1 | 1 | 6.687 |
| 9 | -1 | 0 | -1 | 6.159 |
| 10 | 1 | 0 | -1 | 7.214 |
| 11 | -1 | 0 | 1 | 5.556 |
| 12 | 1 | 0 | 1 | 6.762 |
| 13 | 0 | 0 | 0 | 7.666 |
| 14 | 0 | 0 | 0 | 7.515 |
| 15 | 0 | 0 | 0 | 7.591 |

Supplementary Table S7. Variance analysis of response surface analysis results.

| Term | DF | SS | Adj SS | Adj MS | F | P | Significant |
| --- | --- | --- | --- | --- | --- | --- | --- |
| [Regression](https://fanyi.so.com/#regression) | 9 | 0.001074 | 0.001074 | 0.000119 | 13.71435 | 0.005 | * |
| Linear | 3 | 0.000553 | 0.000553 | 0.000184 | 21.2069 | 0.0028 | ­- |
| Quadratic | 3 | 0.00051 | 0.001047 | 0.00017 | 19.553 | 0.0034 | * |
| Interaction | 3 | 0.00001 | 0.00001 | 3.333E-06 | 0.383142 | 0.7702 | ­- |
| Residual error | 5 | 0.000044 | 0.000044 | 0.0000087 | ­- | ­- | ­- |
| Lack of fit | 3 | 0.000042 | 0.000042 | 0.000047 | 13.83333 | 0.0682 | ­- |
| Pure error | 2 | 0.000002 | 0.000002 | 0.000001 | ­- | ­- | ­- |
| Total | 14 | 0.001117 | ­- | ­- | ­- | ­- | ­- |
| R^2^ | ­ | 0.9611 | 0.8910 | ­- | ­- | ­- | ­- |

Supplementary Table S8. Parameter estimation

| Factors | Coefficient | Coefficient standard error | T | P | Significant |
| --- | --- | --- | --- | --- | --- |
| X1 | 0.00525 | 0.0010428 | 5.0344 | 0.0040 | ** |
| X2 | 0.00525 | 0.0010428 | 5.0344 | 0.0040 | ** |
| X3 | -0.00375 | 0.0010428 | -3.596 | 0.0156 | * |
| X1*X1 | -0.00925 | 0.001535 | -6.026 | 0.0018 | ** |
| X1*X2 | -3.47E-18 | 0.0014748 | -2.35E-15 | 1.0000 | ­- |
| X1*X3 | 0.00050 | 0.0014748 | 0.339903 | 0.7484 | ­- |
| X2*X2 | -0.00575 | 0.001535 | -3.7459 | 0.0133 | * |
| X2*X3 | -0.00150 | 0.0014748 | -1.0171 | 0.3558 | ­- |
| X3*X3 | -0.00625 | 0.001535 | -4.0716 | 0.0096 | ** |

Supplementary Table S9. Purification of chitosanase.

| Term | crude enzyme | SephadexG-75 |
| --- | --- | --- |
| Total activity/U | 7289 | 2803 |
| Total protein/mg | 2405 | 193 |
| Specific activity（U/mg） | 3.03 | 14.52 |
| Fold of purification | 1 | 4.79 |
| Recovery rate（%） | 100% | 38.46 |

Supplementary Table S10. The relationship between the degradation reaction rate of chitosan and substration concentration.

| COS（1*10^3^g.L^-1^) | 0.5 | 1.0 | 1.5 | 2.0 | 3.0 |
| --- | --- | --- | --- | --- | --- |
| V (mg.L^-1^.min^-1^) | 1.0 | 2.0 | 3.0 | 3.9 | 5.9 |
| 1/Cs (L.g^-1^) | 2.0 | 1.0 | 0.7 | 0.5 | 0.3 |
| 1/V (min.L.g^-1^) | 14.5 | 9.3 | 6.4 | 5.4 | 4.4 |

Supplementary Table S11. Statistics Supplementary Table of sequencing-data.

| Statistics of PacBio raw data |  |
| --- | --- |
| Total reads num | 161641 |
| Total bases | 1702336386 |
| N50 | 13071 |
| Average length  Average coverage | 10531  275 |
| Statistics of Illumina high quality data |  |
| Total reads num | 11609550 |
| Total bases | 1741432500 |
| Read length | 150 |
| Q20(%) | 91.13 |
| Q30(%) | 80.80 |
| Average coverage | 282 |

Supplementary Table S12. Assembled statistics of *Mitsuaria sp.*Strain C4

| Name | C4 |
| --- | --- |
| No. of all scaffolds | 1 |
| Bases in all scaffolds | 6,188,406 |
| G+C content (%) | 68.4 |
| N rate | 0 |

Supplementary Table S13. Genome characteristics of C4 and other *Mitsuaria sp..*

| Strains | Genome size (Mb) | GC content (%) | Protein-coding genes |
| --- | --- | --- | --- |
| *Mitsuaria sp.* C4 | 6.18 | 68.40 | 5,268 |
| *Mitsuaria sp.* 7 | 6.09 | 68.28 | 5,282 |
| *Mitsuaria sp.* BK037 | 5.85 | 70.00 | 4,924 |
| *Mitsuaria sp.* BK041 | 5.86 | 70.00 | 4,921 |
| *Mitsuaria sp.* BK045 | 5.86 | 70.00 | 4,919 |
| *Mitsuaria sp.* HWN-4 | 5.74 | 69.50 | 4,895 |
| *Mitsuaria sp.* PDC51 | 5.85 | 70.00 | 4,946 |
| *Mitsuaria sp.* TWR114 | 5.63 | 69.50 | 4,893 |
| *Mitsuaria sp.* WAJ17 | 5.31 | 68.00 | 4,608 |
| *M. chitosanitabida* | 5.82 | 69.90 | 5,029 |
| *M. noduli* | 6.50 | 68.50 | 5,645 |

Supplementary Table S14. Statistical analysis of non-coding RNAs.

| Class | number | totalLen | meanLen |
| --- | --- | --- | --- |
| Cis-reg | 5 | 478 | 95.6 |
| Cis-reg-riboswitch | 9 | 1383 | 153.6666667 |
| Gene | 3 | 651 | 217 |
| Gene-rRNA | 12 | 18091 | 1507.583333 |
| Gene-ribozyme | 1 | 336 | 336 |
| Gene-sRNA | 3 | 473 | 157.6666667 |
| Gene-tRNA | 62 | 4679 | 75.46774194 |

Supplementary Table S15. Repetitive element annotations.

|  | number elements | of occupied | length | Percentage of genome (%) |
| --- | --- | --- | --- | --- |
|  |  |  |  |  |
| Retroelements | 54 | 5328 | bp | 0.09 |
| SINEs: | 18 | 945 | bp | 0.02 |
| Penelope | 0 | 0 | bp | 0 |
| LINEs: | 10 | 1117 | bp | 0.02 |
| CRE/SLACS | 1 | 450 | bp | 0.01 |
| L2/CR1/Rex | 1 | 35 | bp | 0 |
| R1/LOA/Jockey | 3 | 232 | bp | 0 |
| R2/R4/NeSL | 0 | 0 | bp | 0 |
| RTE/Bov-B | 2 | 160 | bp | 0 |
| L1/CIN4 | 1 | 62 | bp | 0 |
| LTR elements: | 26 | 3266 | bp | 0.05 |
| BEL/Pao | 2 | 181 | bp | 0 |
| Ty1/Copia | 12 | 1573 | bp | 0.03 |
| Gypsy/DIRS1 | 9 | 1164 | bp | 0.02 |
| Retroviral | 2 | 281 | bp | 0 |
| DNA transposons | 16 | 2130 | bp | 0.03 |
| hobo-Activator | 4 | 444 | bp | 0.01 |
| Tc1-IS630-Pogo | 1 | 42 | bp | 0 |
| Tourist/Harbinger | 1 | 102 | bp | 0 |
| Unclassified: | 7 | 3558 | bp | 0.06 |
| Small RNA: | 48 | 9922 | bp | 0.16 |
| Satellites: | 1 | 47 | bp | 0 |
| Simple repeats: | 1939 | 99159 | bp | 1.6 |
| Low complexity: | 43 | 2200 | bp | 0.04 |

Supplementary Table S16. Annotation statistics for the C4 genome.

| Annotation statistics for the genome | | Number | Percent (%) |
| --- | --- | --- | --- |
|  | Total protein | 5,268 |  |
|  | eggNOG | 4,460 | 84.66 |
|  | GO | 1,701 | 32.29 |
|  | COG | 4,209 | 79.89 |
|  | KEGG | 1,881 | 35.71 |
|  | In at least one database | 4,553 | 86.42 |
