# Supplementary material for: Chitosan Oligosaccharide Production Potential of Mitsuaria sp. C4 and Its Whole-Genome Sequencing
Source: Front Microbiol. 2021 Aug 5;12:695571. doi: 10.3389/fmicb.2021.695571 (PMC8374441; doi:10.3389/fmicb.2021.695571)
Supplement: Supplementary file 2 [file Table_2.DOC]

**GRAPHICAL ABSTRACT**


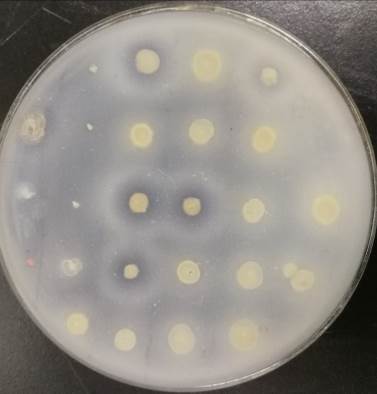

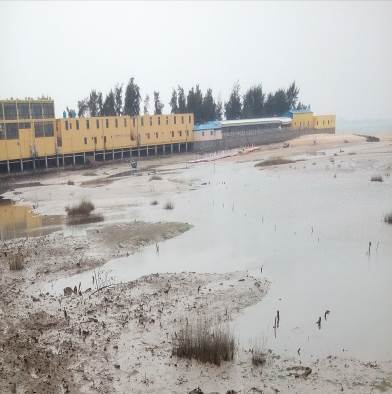

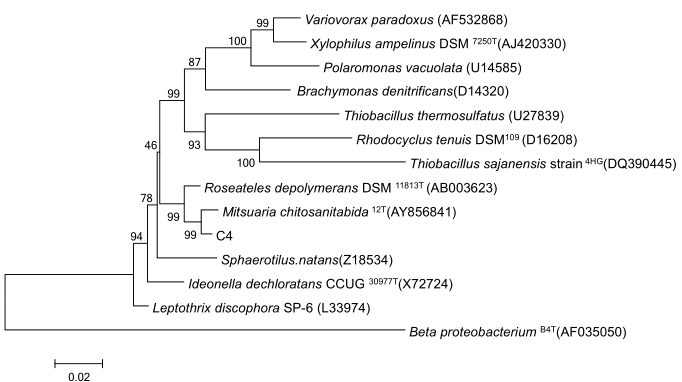

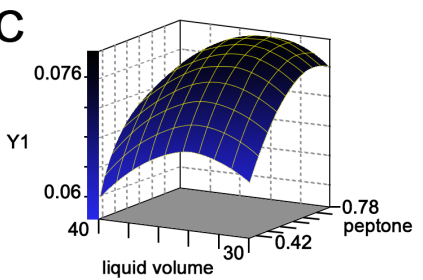

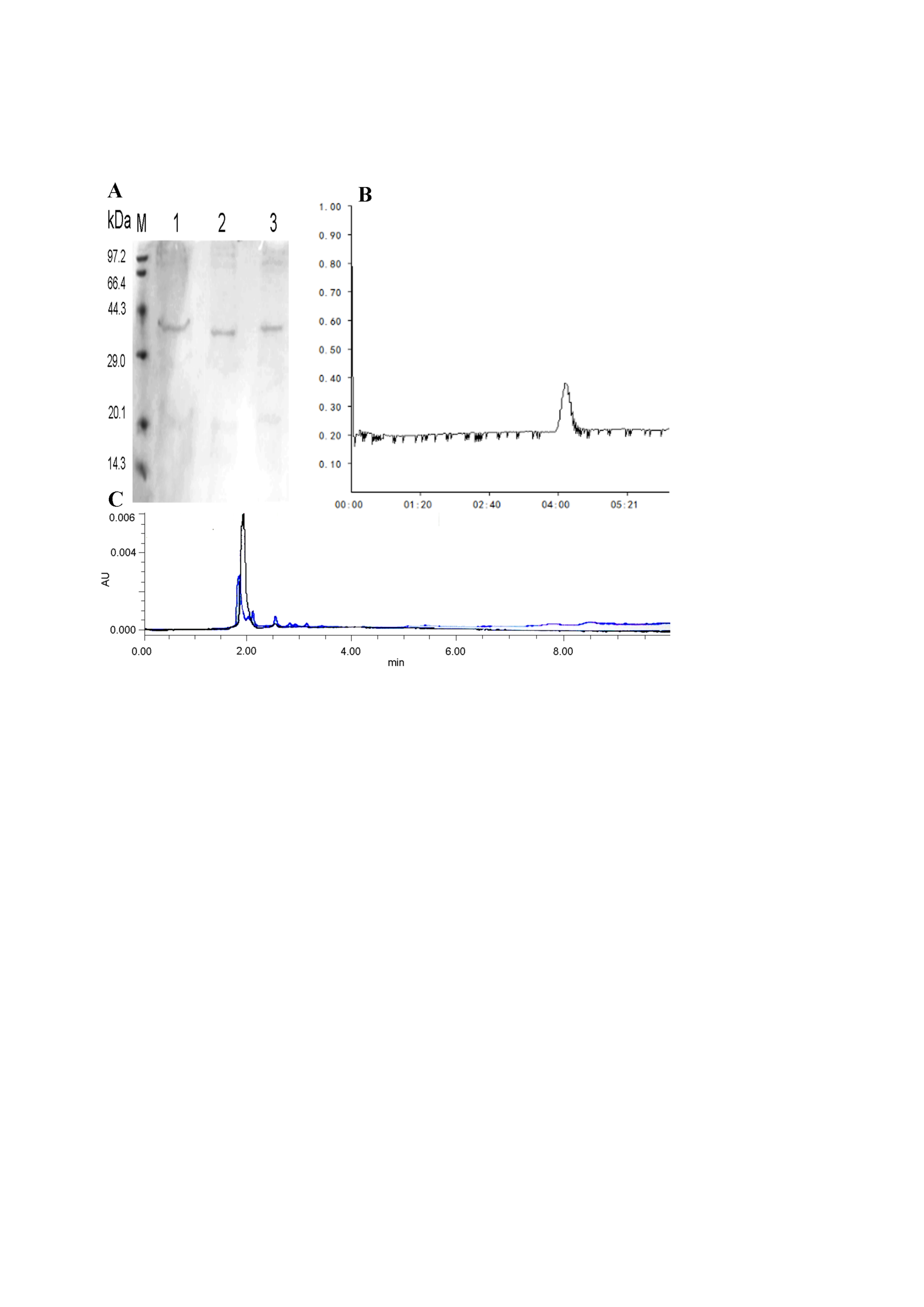

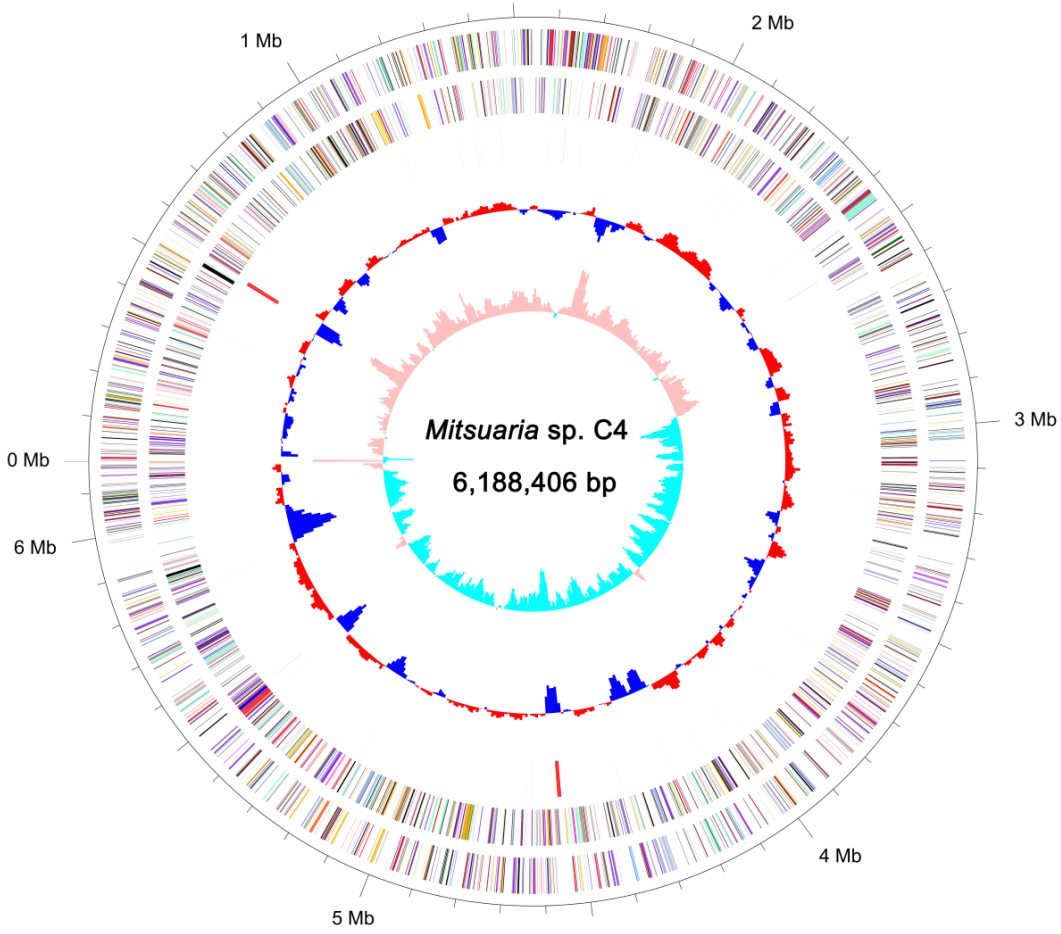


**Sampling site：**[**Xisha bay,**](https://xueshu.baidu.com/usercenter/paper/show?paperid=04dde2ca03ea3fc8597efe0f48154dce&site=xueshu_se&sc_from=AQNU) **Quanzhou, Fujian province, China**

**Screening and identification**

**Optimized production**

**Purification**

**Characterization**

pH- and thermo-stability

substrate specificity, kinetic constants

chitosan hydrolysates

metal ions-stability

**Whole-genome sequencing**
