# Supplementary material for: Chitosan Oligosaccharide Production Potential of Mitsuaria sp. C4 and Its Whole-Genome Sequencing
Source: Front Microbiol. 2021 Aug 5;12:695571. doi: 10.3389/fmicb.2021.695571 (PMC8374441; doi:10.3389/fmicb.2021.695571)
Supplement: Supplementary file 3 [file Table_3.DOCX]

**Supplementary Figures**


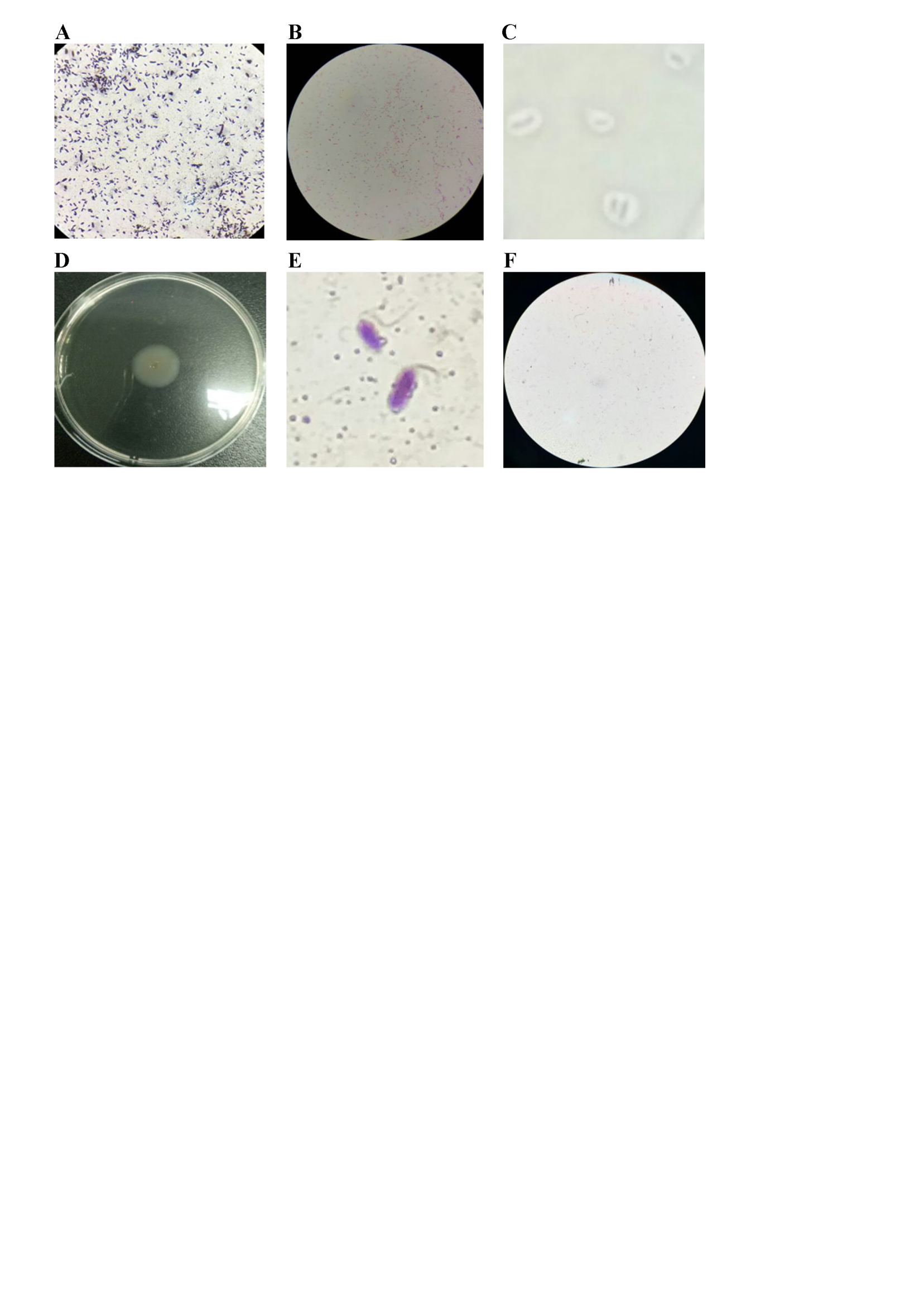


Supplementary Figure S1. Identification of C4 by morphology and biochemistry. (A) Basic feature of morphology; (B) Gram’s staining; (C) Capsule staining; (D) Puncture inoculation; (E) Flagella staining; (F) Spore staining.

A B

C D

E F

Supplementary Figure S2. Effect of temperature, pH, and metal ion on chitosanase activity and stability. (A) Effect of temperature on chitosanase activity; (B) Effect of temperature on chitosanase stability; (C) Effect of pH on chitosanase activity; (D) Effect of pH on chitosanase stability; (E) Effect of metal ion on chitosanase activity; (F) Substrate specificity of the chitosanase.

A B


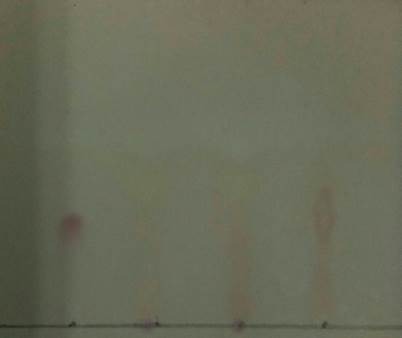
 **S1 S2 S3 S4**

C D


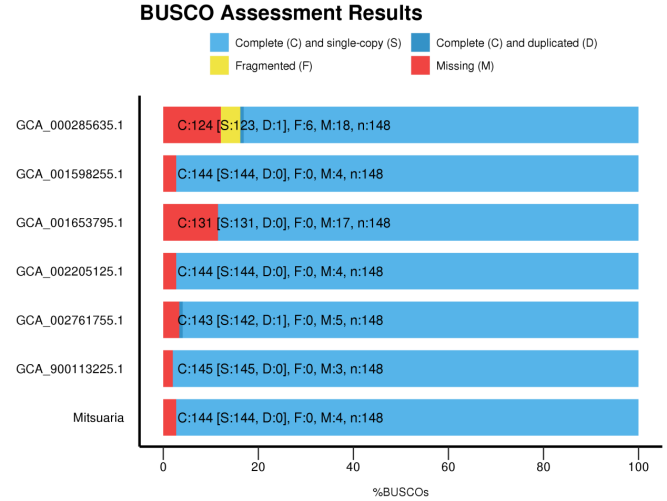

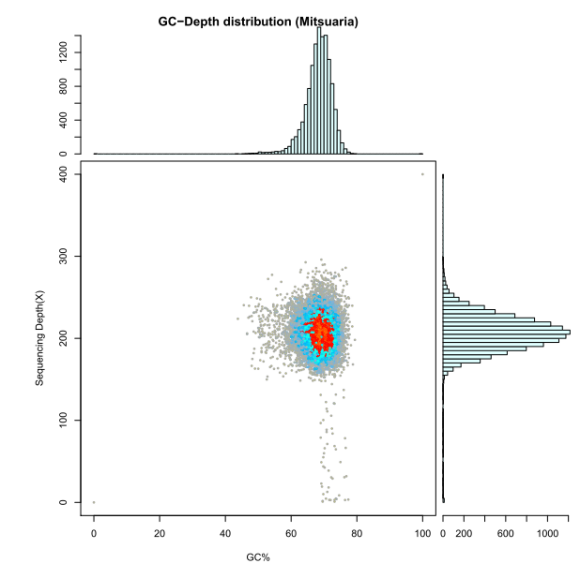


Supplementary Figure S3. Analysis of chitosan hydrolysate and *Mitsuaria sp.* genome characteristic. (A) TLC plate of the hydrolysis of different ratio of COS and crude enzyme solution.S1: 1/0; S2: 10/1, S3:5/1; S4:1/1; (B) Double reciprocal plots of chitosanase-polysaccharide chitosan reaction; (C) Correlation analysis of GC content and Depth; (D) Comparison results of Mitsuaria sp. genomic integrity.

A B


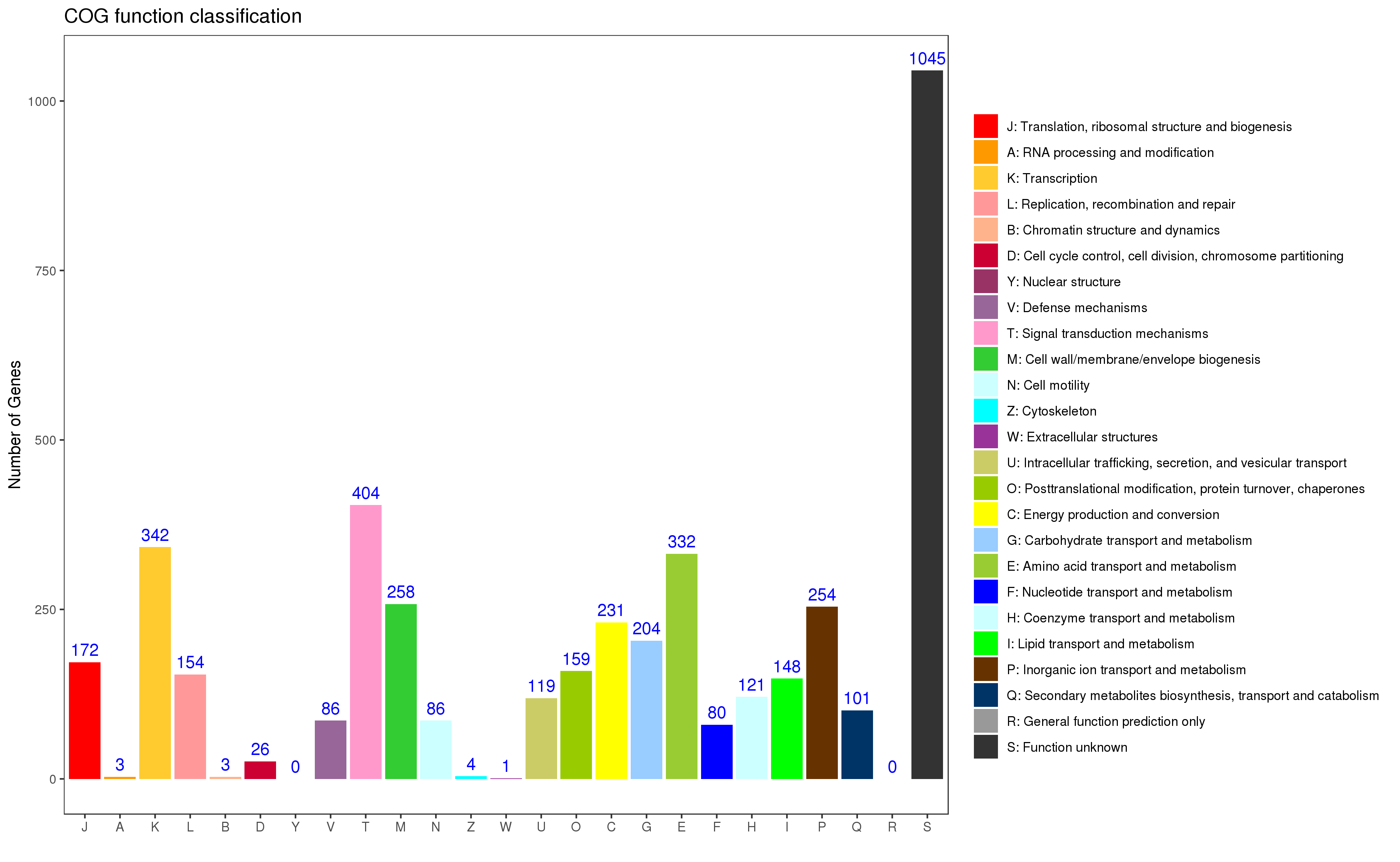

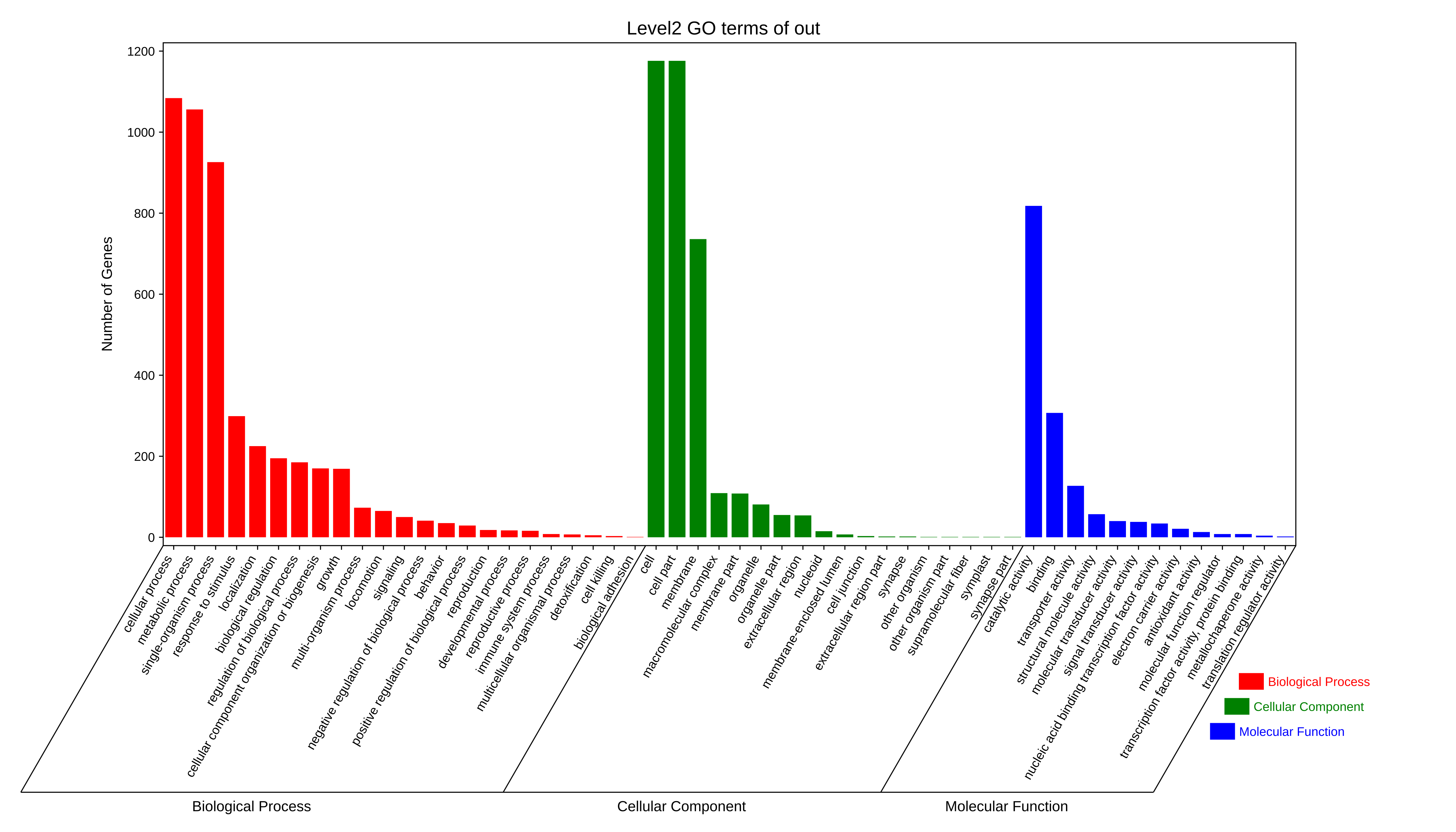
C D


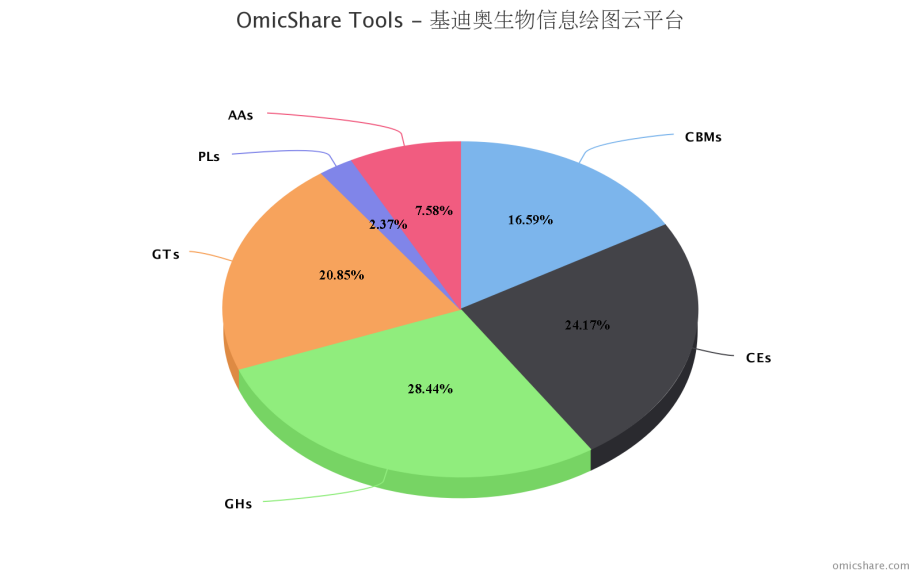

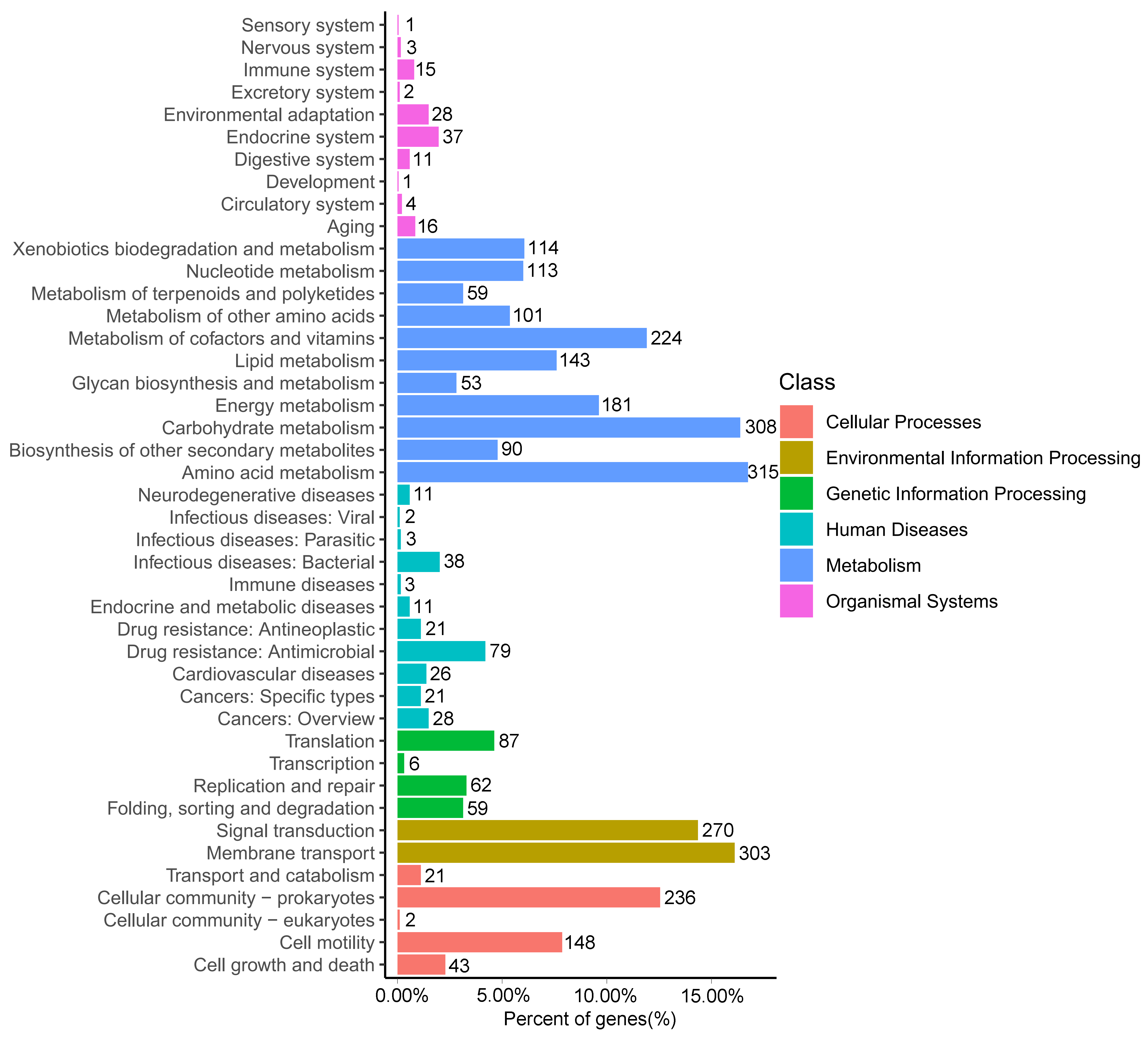


Supplementary Figure S4. Functional categories of *Mitsuaria sp.* C4 annotated by GO (A), COG (B), KEGG (C) and CAZy (D).
